# Cigarette smoke differentially modulates dendritic cell maturation and function in time

**DOI:** 10.1186/s12931-015-0291-6

**Published:** 2015-10-24

**Authors:** Masoumeh Ezzati Givi, Gert Folkerts, Gerry T. M. Wagenaar, Frank A. Redegeld, Esmaeil Mortaz

**Affiliations:** Division of Pharmacology, Utrecht Institute for Pharmaceutical Sciences, Faculty of Science, Utrecht University, PO BOX 80082, 3508 TB Utrecht, The Netherlands; Department of Pediatrics, Division of Neonatology, Leiden University Medical Center, Leiden, The Netherlands; Chronic Respiratory Diseases Research Center and National Research Institute of Tuberculosis and Lung Diseases (NRITLD), Department of Immunology, Shahid Beheshti University of Medical Sciences, Tehran, Iran; Airways Disease Section, National Heart and Lung Institute, Imperial College London, London, UK; Department of Pharmacology and Toxicology, Faculty of Veterinary Medicine, Shahid Chamran University, Ahvaz, Iran

**Keywords:** Dendritic cells, Cigarette smoke, Bone marrow, COPD

## Abstract

**Background:**

Dendritic cells (DCs) as professional antigen presenting cells (APCs) play a critical role in the regulation of host immune responses. DCs evolve from immature, antigen-capturing cells, to mature antigen-presenting cells. The relative contribution of DCs to cigarette smoke-induced inflammation is not well documented. In the current study, we investigated a modulatory effect of cigarette smoke extract (CSE) on differentiation, maturation and function of DCs.

**Methods:**

Primary murine DCs were grown from bone marrow cells with GM-CSF. Development of DC was analyzed by expression of CD11c, MHCII, CD86, CD40 and CD83 using flow cytometry. Murine DC’s and human L428 cells were co-cultured with CSE for various periods of time. Functional activity was analyzed by measuring FITC-dextran uptake, cytokine production and the ability to stimulate T cell activation in a mixed lymphocyte reaction.

**Results:**

Our results show that short-term CSE stimulation (~24 h) influence the maturation status of newly differentiated and immature DCs towards more mature cells as revealed by upregulation of MHCII, CD83, CD86, CD40, reduction in antigen up-take capacity and enhanced secretion of pro-inflammatory (IL-12, IL-6 and TNF-α) cytokines. Interestingly, long-term CSE exposure, time- and concentration-dependently, suppressed the development of functional DCs. This suppression was demonstrated by a decline in CD11c/MHCII, CD83, CD86 and CD40 expression, the production of cytokines and ability to stimulate T lymphocytes. Moreover, CSE significantly suppressed the endocytosis function of mouse DCs which was not due to diminished DC viability. Similar to mouse DCs, long-term co-culturing of the human L428 DC cell line with CSE time-dependently suppressed the expression of CD54.

**Conclusions:**

The present study provides evidence that CSE modulates DC-mediated immune responses via affecting both the function and maturation of DCs. The suppressive effects of cigarette smoke on DC function might lead to impaired immune responses to various infections.

**Electronic supplementary material:**

The online version of this article (doi:10.1186/s12931-015-0291-6) contains supplementary material, which is available to authorized users.

## Background

Cigarette smoking is the main risk factor for the development of inflammatory lung disease such as chronic obstructive pulmonary disease (COPD) which is a slowly progressive disease [1]. The lung inflammatory response to CS exposure is complex and mechanisms initiating this response are still poorly understood. It has been shown that cigarette smoke (CS) contains a complex mixture of chemicals, bacterial and fungal components including LPS [2] that are capable of exerting immune-modulating effects. Thus, understanding in detail the mechanisms underlying inflammatory process induced by CS may lead to better therapeutic approaches in COPD. Many inflammatory cells and their mediators, both of the innate and adaptive immune system, play a role in the pathogenesis of disease [3]. Macrophages, neutrophils and lymphocyte are the cells usually considered the prime effector cells in immune response to CS, but recently DCs have been suggested to be a potentially important new player/orchestrator of the pattern of inflammation induced by CS [4].

DCs are essential antigen-presenting cells (APCs) and orchestrate innate inflammatory responses and adaptive immunity through activation of T cells via direct cell-cell interactions and/or cytokine production [5, 6]. In both humans and mice there are several subtypes of DC, as characterized by surface markers and function. Generally, DC subsets arise from bone marrow (BM) precursors that colonize peripheral tissues through blood or the lymphatic system [6–9]. Pulmonary DCs distribute in sub-epithelial, interstitial and pleural compartments where they usually exist as immature antigen presenting cells. Immature DCs are efficient in antigen uptake, but during DC maturation antigen uptake ability decreases as the antigen presenting ability is enhanced. MHCII molecules present the first classical signal in the process of antigen presentation, and co-stimulatory molecules such as CD86, CD40 represent the second signal. Since DCs are so well equipped to initiate adaptive immune responses, they are considered prime targets for modulating immune responses.

The number of DCs *in vivo* is low compared with most other immune cells, and their isolation in sufficient numbers for comprehensive studies is laborious and expensive. Therefore, the majority of studies use *in vitro* generated DCs from bone marrow cells or blood monocyte [10–13].

Studies using bone marrow and monocyte-derived DCs exposed to varying concentrations of cigarette smoke extract (CSE) and nicotine yielded contrasting results with respect to the effects on DC function [14–17]. The importance of DCs in maintaining host immunity led us to further investigate whether DCs are affected by exposure to CS.

## Methods

### Preparation of CSE

CSE was produced following the method as described before [18]. Briefly CSE was generated by the burning of commercially available Lucky Strike cigarettes without filter (British–American Tobacco, Groningen, The Netherlands), using the TE-10z smoking machine (Teague Enterprises, Davis, CA, USA), which is programmed to smoke cigarettes according to the Federal Trade Commission protocol (35-ml puff volume drawn for 2 s, once per minute). This machine was used to direct main- and side-stream smoke from one cigarette through a 5-ml culture medium (RPMI without phenol red). Hereafter, absorbance was measured with a spectrophotometer, and the media were standardized to a standard curve of CSE concentration against absorbance at 320 nm. The pH of the resultant extract was titrated to pH 7.4 and diluted with medium. This concentration (optical density (OD) = 4.0) was serially diluted with untreated media to 0.5–3 % OD and used in the indicated experiments.

### Cell preparation and experimental design

#### Culture of mouse BMDCs

**Fig. 1:**
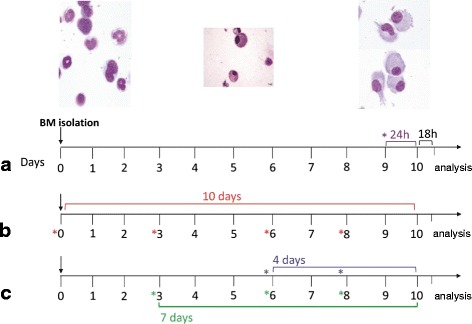
Experimental design diagram: Generation of BM-derived DCs with GM-CSF in presence or absence of CSE during 10 days. The cultures were re-cultured with fresh medium containing GM-CSF (20 ng/ml) at days 0, 3, 6 and 8. **a** In order to investigate the acute effects of CSE on the full maturation of DCs, cells were incubated with CSE (0.05–1.5 %) or LPS (0.01–1 μg/ml, as positive control) for the final 24 h of culture period or the next 18 h after the culture period. **b** To evaluate the prolonged immune modulatory effect of CSE on DCs differentiation process, BM cells were cultured in the presence or absence of CSE (0–1.5 %) or LPS (0.01–1 μg/ml) from day 0 for 10 days. **c** To investigate timing effect of CS exposure in the differentiation of BM precursors to DCs, cells were co-cultured with CSE (1.5 %) or LPS (100 ng/ml) at various time points of culture from day 3 or day 6 for 7 or 4 days in parallel experiments. *Indicate administration of CSE or LPS

The method for generating BM-derived DCs was adapted from that described by Lutz and coworkers [19] with slight modifications. The use of animals in these studies was approved by the animal ethics committee of the Utrecht University. BM cells isolated from BALB/c mice (4-to 12-week-old) and were cultured in complete RPMI 1640 supplemented with GM-CSF 200u/ml (=20 ng/ml) for 10 days. In order to investigate how CSE influenced full maturation of DCs, the non-adherent cells were incubated with CSE (0.05–1.5 %) or LPS (0.01–1 μg/ml, as positive control) for the final 24 h of culture period or the next 18 h after the culture period (Fig. 1a). Subsequently, to evaluate the immune modulatory effect of CSE on DCs differentiation process, BM cells were cultured in the presence or absence of CSE (0–1.5 %) or LPS (0.01–1 μg/ml) from day 0 for 10 days (Fig. 1b). To investigate the effect of timing of the CSE exposure in the differentiation of BM precursors to DCs, cells were co-cultured with CSE (1.5 %) or LPS (100 ng/ml) at various time points of culture at from day 3 or day 6 for 7 or 4 days in parallel experiments (Fig. 1c). Non adherent and loosely adherent cells were harvested for analysis. DCs responses were assayed using ELISA (cytokine production) and flow cytometry analysis (surface marker expression). Nontoxic effects of up to 1.5 % concentration of CSE was found since viability were consistently established to be >95 % (trypan blue exclusion).

#### Mixed lymphocyte reaction

To assess the function of CSE and LPS-stimulated BMDCs the mixed lymphocyte reaction (MLR) was used. Briefly, spleens from D011.10 TAC (kindly provided by dr Janneke Samson, EUR, Rotterdam, the Netherlands) were prepared and CD4 + KJ1.26+ T cells were isolated using a CELLection Biotin Binder kit isolation kit (Life Technologies) according to manufacturer’s instructions. Freshly isolated T cells were stained with 5, 6-carboxy-succinimidyl-fluorescein-ester dye (CFSE) (CellTrace™ CFSE Cell Proliferation Kit, Life Technologies) and co-cultured with BMDCs at a DC:T cell ratio of 1:5, 1:10 and 1:20 in presence of ovalbumin protein in round-bottom 96-well microtiter plates for 72 h. At the end of 72 h, supernatant and cells were collected for cytokine measurement (IL −6, −10, −12p70 and IFN-γ) using a cytometric bead array kit (BD CBA Flex Sets) and T cell proliferation was measured by flow cytometry using a BD FACS CantoII flow cytometer.

#### Culture of L428 cells

The Hodgkin’s disease (HD)-derived cell line L428 most closely resembles a human DCs phenotype and function [20, 21]. Thus, L428 (kindly provided by University of California) was used in this experiment as a human-DC model to determine the long-term effect of CSE on human DCs activation. Cells were maintained at 4 × l06 to 2 × 106 cells/ mL in RPMI-1640 supplemented with 10 % FBS, L-glutamine (2 mM), penicillin (100 U/ml), streptomycin (100 mg/ml), 2-mercaptoethanol (50 μmol/L) and gentamicin (50 μg/mL) at 37 °C in a humidified 5 % CO2 atmosphere. The medium was replenished twice weekly, 24–48 h prior to assay. The viability of these cells was maintained at >95 % (Trypan Blue dye exclusion). These cells have a doubling time of approximately 60 to 84 h. Following the third subconfluent passage, cells were cultured in the presence or absence of CSE (1.5 %) or LPS (100 ng/ml) for periods of 10, 20 and 30 days. At the end of each experimental period, non-adherent and loosely adherent cells were harvested for FACS analysis.

#### Flow cytometry analysis

Surface receptor expression on mouse and L428 DCs was determined by FACS analysis. To this end, cells were washed once with 1x PBS/0.3 % BSA and stained with primary antibodies directly conjugated to fluorochromes for 30 min at 4oC. Dead cells were excluded using annexin-V and 7-AAD (BD Biosciences) viability staining. Live events were acquired on a FACSCanto II flow cytometer (BD Biosciences), and data were analyzed with FACSDiva software (v6.1.2). The following antibodies were used for flow cytometry analysis: PE-Cy7–conjugated anti-mouse CD11c, FITC-conjugated anti–major histocompatibility complex (MHC) class II, PE-conjugated anti-mouse CD86, APC-conjugated anti-mouse-CD40 and -CD83 and PE-conjugated anti-human CD54. All antibodies were purchased from eBioscience or BD Biosciences (San Diego, CA).

#### FITC–dextran uptake

To assess DC endocytic activity, BMDCs were suspended in RPMI 1640 supplemented with 10 % FCS and incubated with 1 mg/ml of FITC–dextran (Fluorescein isothiocyanate-labeled dextrans) (Mr = 40,500; Sigma Aldrich, the Netherlands) for 30 min at 4 or 37 °C. Cells were washed three times with ice-cold PBS, 0.1 % BSA and 0.01 % NaN3, and labeled on ice with appropriate mAb. The uptake was calculated as the change in mean fluorescence intensity (MFI) between cell samples incubated at 37 and 4 °C.

#### Cytokine assay

The inflammatory cytokines; IL-12, IL-6 and TNF-α, were quantified at the protein level in supernatants of BMDCs using ELISA kits (BD Pharmingen) according to the manufacturer’s instructions.

#### Statistical analysis

Experimental results are expressed as mean ± S.E.M. Results were tested statistically by a one-way ANOVA followed by Newman-Keuls test for comparing all pairs of groups or two-tailed, non-paired, student’s *t*-test. Analyses were performed using GraphPad Prism (version 5.0). Results were considered statistically significant when *P* < 0.05.

## Results

### CSE effects on DC maturation during short-term and long-term incubation

**Fig. 2:**
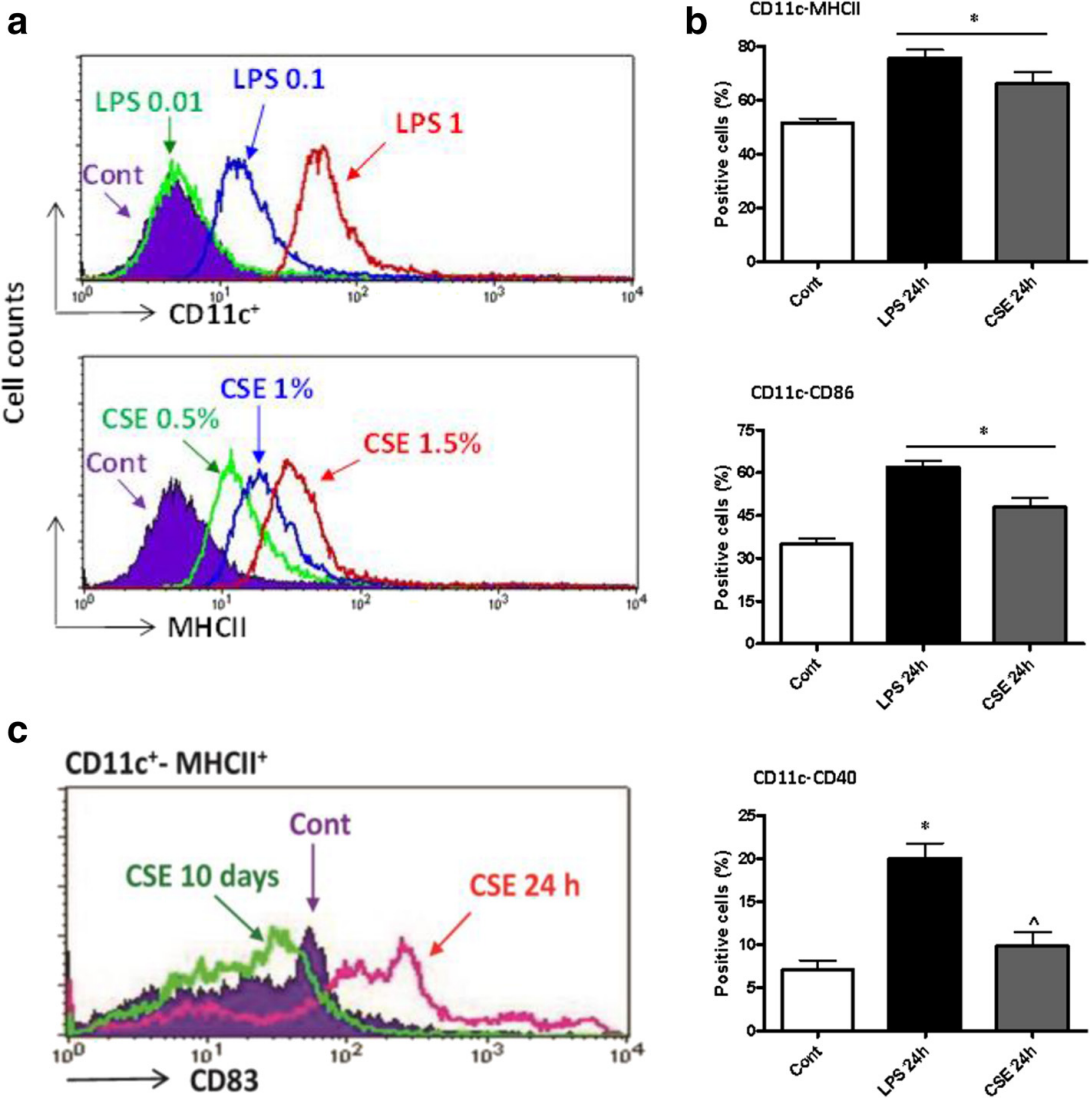
CSE induces DC maturation during short-term stimulation. Representative histograms (left panel) showing the expression of the cell surface DC maturation markers CD11c, MHCII and CD83. Bar graphs (right panel) represent expression of CD11c-MHCII and co-stimulatory molecules CD86, CD40 as percentage of positive DCs. BMDCs were cultured with (**a**) CSE (0.05–1.5 %) or LPS (0.01–1 μg/ml, as a positive control) for 18 h, (**b**) CSE (1.5 %) or LPS (1 μg) for final 24 h and (**c**) CSE (1.5 %) for 10 days of culture period. Data represent mean ± S.E.M. *P < 0.05 significantly different compared to control, ^ *P* < 0.05 significantly different compared to LPS 24 h (*n* = 7)

In order to investigate the acute effects of CSE on the full maturation of DCs, BMDCs were incubated with CSE (0.05–1.5 %) or LPS (0.01–1 μg/ml, as positive control) for 18 h after 10 days of culture (Fig. 1a). CSE increased CD11c and MHCII expression, concentration dependently (Fig. 2a). Further experiments were performed with a CSE concentration of 1.5 % since higher concentrations were toxic.

Next, we examined the effect of short-term culturing of DCs with CSE for the final 24 h of the 10 days culturing period (Fig. 1a). Consistent with an increased maturation, CSE induced the expression of CD11c, MHCII (Fig. 2b), the co-stimulatory molecules CD86, CD40 (Fig. 2b) and CD83 (Fig. 2c). To test the long-term CSE effects on DC maturation, DC precursors were cultured in the presence of CSE for 10 days (Fig. 1b). In contrast to short exposure time, long-term incubation with CSE resulted in a suppression of CD11c-MHCII/CD83 expression (Fig. 2c).

### CSE increased the developing of defective and silent DCs during long-term stimulation

**Fig. 3:**
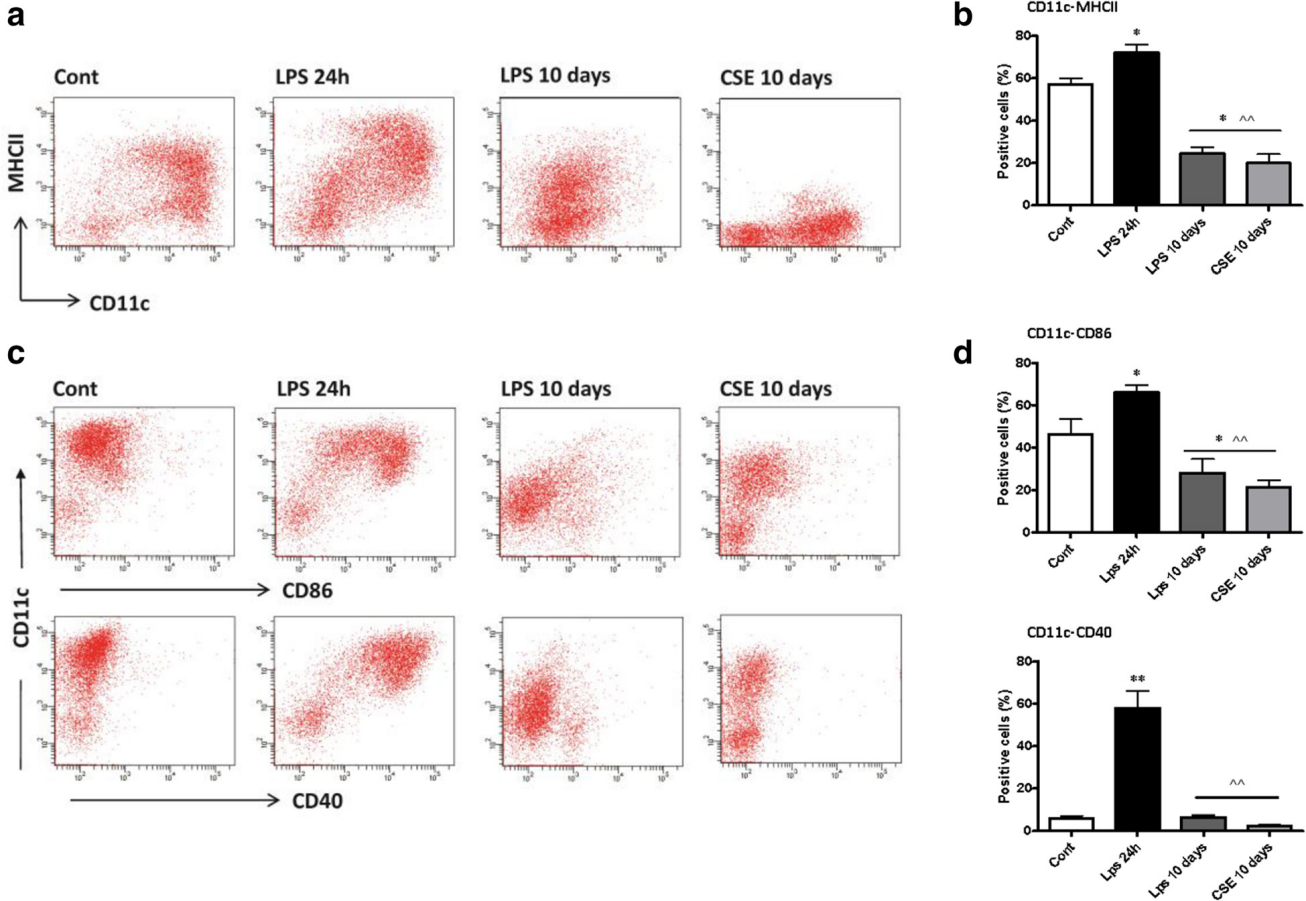
Long-term continuous exposure to CSE suppresses the development of functional DCs from BM precursors. BM precursors were cultured in the presence or absence of CSE (1.5 %) or LPS (100 ng/ml, as positive control) continuously with every feeding day. Representative dot plots, (panels **a** and **c**) and bars (panels **b** and **d**) show percentages of DCs positive for CD11c-MHCII (**a**, **b**) CD11c-CD86 and -CD40 (**c**, **d**). Data in A and C show one representative experiment of seven. Data represent mean ± S.E.M. *P < 0.05, **P < 0.01 significantly different compared to control and ^^ *P* < 0.01 significantly different compared to LPS 24 h

To examine whether CSE influenced the development of DCs from BM precursors, isolated BM cells were cultured in the presence or absence of CSE or LPS continuously (Fig. 1b). At the end of *day* 10, the non-adherent and loosely adherent cells were analyzed for the expression of cell surface markers. Continuous exposure to CSE or LPS during DC maturation significantly down regulated the expression of CD11c-MHCII (Fig. 3a and b), and CD40, CD86 markers (Fig. 3c and d). Next, we tested intracellular expression of these markers and did not find any signs of internalization of these receptors (data not shown). At all-time points of culture, total cell numbers generated per dish under CSE or LPS condition were not reduced, which indicates that CSE does not modulate the expansion of DC precursor cells but rather their maturation.

Furthermore, expression of other monocyte markers (CD14), macrophages (F4/80) were not detectable (data not shown). No significant changes in the percentage of apoptotic and necrotic DCs were found as determined by using annexin-V and 7-AAD viability staining.

### CSE induces DC suppression in a concentration- and time-dependent manner

**Fig. 4:**
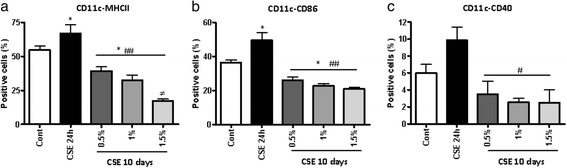
CSE suppresses DC maturation during long-term stimulation in a concentration-dependent manner. BM precursors were cultured in presence or absence of CSE (0.5–1.5 %) or LPS (0.01–1 μg/ml, as positive control) continuously with every re-culturing. Representative data show percentages of DCs positive for CD11c-MHCII (**a**) CD11c-CD86 (**b**) and -CD40 (**c**). Data represent mean ± S.E.M (*n* = 7). *P < 0.05 significantly different compared to control,# *P* < 0.05, ## *P* < 0.01significantly different compared to CSE 24 h and ≠*P* < 0.05 significantly different compared to CSE 0.5 %

To determine whether the suppressive effect of CSE on DC differentiation is concentration dependent, DC precursors were cultured prolonged in presence of different concentrations of CSE (0.5–1.5 %) at multiple time points of culture. The maturation of DCs was suppressed by CSE as revealed by down regulation of CD11c, MHCII, CD86 and CD40 molecules in concentration-dependent manner (Fig. 4). In the search for specific CS constituents, which could be responsible for suppression of DCs maturation and development, nicotine and acrolein were tested but none of them mimicked the effect of the complete CSE (data was not shown).

However, LPS as an important bioactive component of CSE caused a similar suppression of DC maturation in long-term co-culture experiments (Fig. 3).

**Fig. 5:**
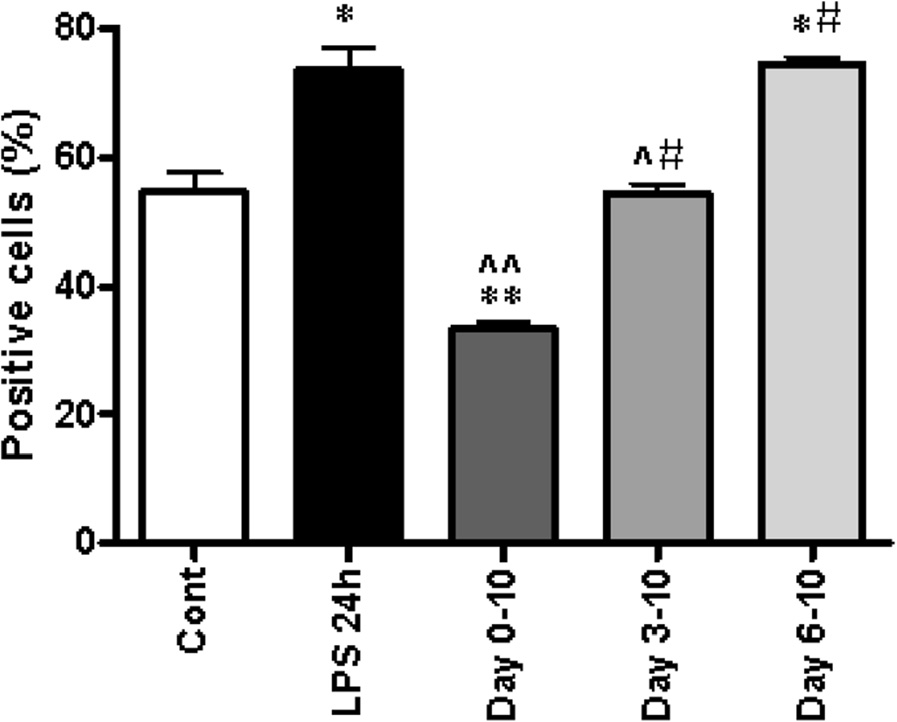
CSE affects the DCs maturation time dependently. Data show the percentage of CD11c-MHCII-positive DCs. BMDCs were cultured in with CSE (1.5 %) or LPS (100 ng/ml) continuously for different periods of time. Data represent mean ± S.E.M (*n* = 4). *P < 0.05, **P < 0.01 significantly different compared to control, ^ *P* < 0.05, ^^*P* < 0.01 significantly different compared to LPS 24 h, # *P* < 0.05significantly different compared to CSE day 0–10 and 6–10

Subsequently, DC precursors were cultured with CSE continuously for different time periods as described in Methods (Fig. 1b and c). Co-culture of DCs from *day* 0–10 with CSE resulted in low expression of cell surface markers (*P* < 0.05). However, DC undergoing CSE exposure from *day* 3 did not show a difference in CD11c-MHCII expression (Fig. 5). Interestingly, incubation from *day* 6 significantly increased the expression of cell surface markers to the same level as the positive control (LPS) (Fig. 5). This means, that the effects of CSE are differentially regulated in time.

### Time-dependent effect of CSE on cytokine release by DCs

**Fig. 6:**
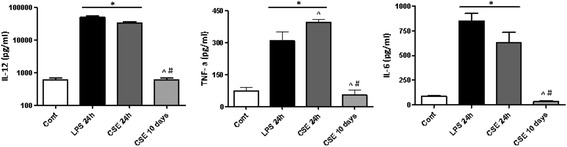
CSE suppresses the DCs cytokine responsiveness during long-term stimulation. The culture supernatant from 10 days or last 24 h CSE-treated DCs were harvested and IL-12, IL-6 and TNF- α cytokines production measured by ELISA. Data represent mean ± S.E.M (*n* = 7). *P < 0.05 significantly different compared to control, ^ *P* < 0.05 significantly different compared to LPS 24 h and # *P* < 0.05 significantly different compared to CSE 24 h

Next, we investigated whether the suppressive effect of CSE on DCs maturation affected the cytokine production. As shown in Fig. 6, the supernatant of DC differentiated in the presence of CSE for 10 days showed no IL-12, TNF-α and IL-6 production. In contrast, short-term co-culturing of DCs with CSE or LPS for the final 24 h resulted in a significant release of these cytokines.

### CSE suppressed DC function during prolonged stimulation for 10 days

**Fig. 7:**
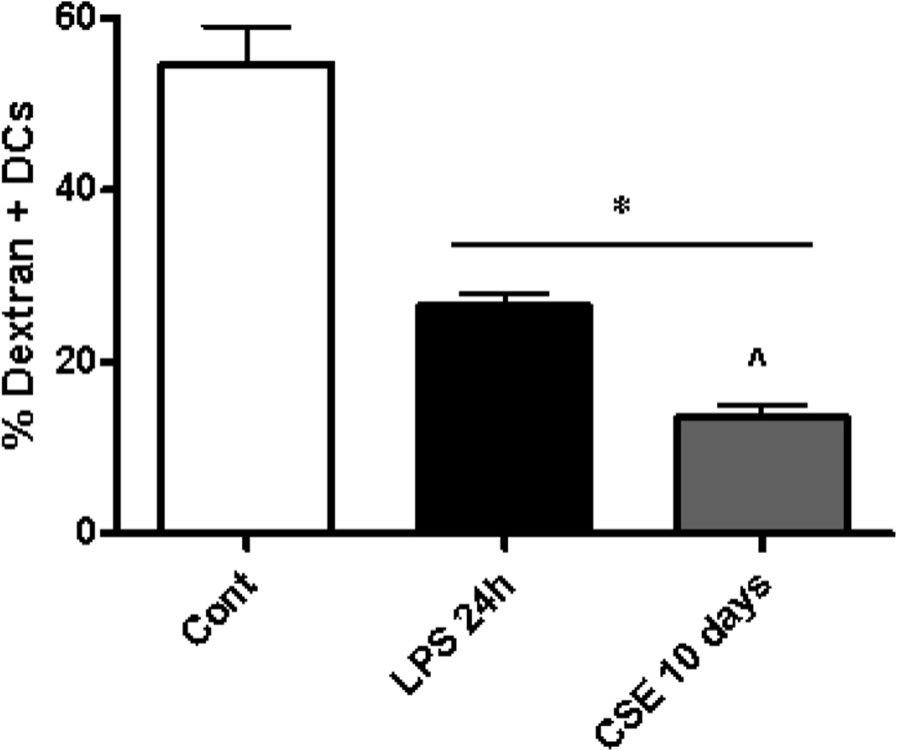
CSE suppresses the FITC-dextran uptake by DCs. The endocytosis activity of DCs was measured by the FITC-dextran uptake. Data represent mean ± S.E.M. (*n* = 4) *P < 0.05 significantly different compared to control and ^ *P* < 0.05 significantly different compared to LPS 24 h

Immature DCs efficiently take up antigens and this function is suppressed after maturation [22]. After co-culture with CSE during 10 days, the endocytosis activity of DCs was measured by FITC-dextran uptake. The FITC-dextran uptake was reduced in DCs that were differentiated in the presence of CSE (Fig. 7). Indeed, maturation of DC’s for 24 h with LPS as a positive control, showed a decrease in FITC-dextran uptake. In all experiments, treatments did not affect cell viability (data not shown).

**Fig. 8:**
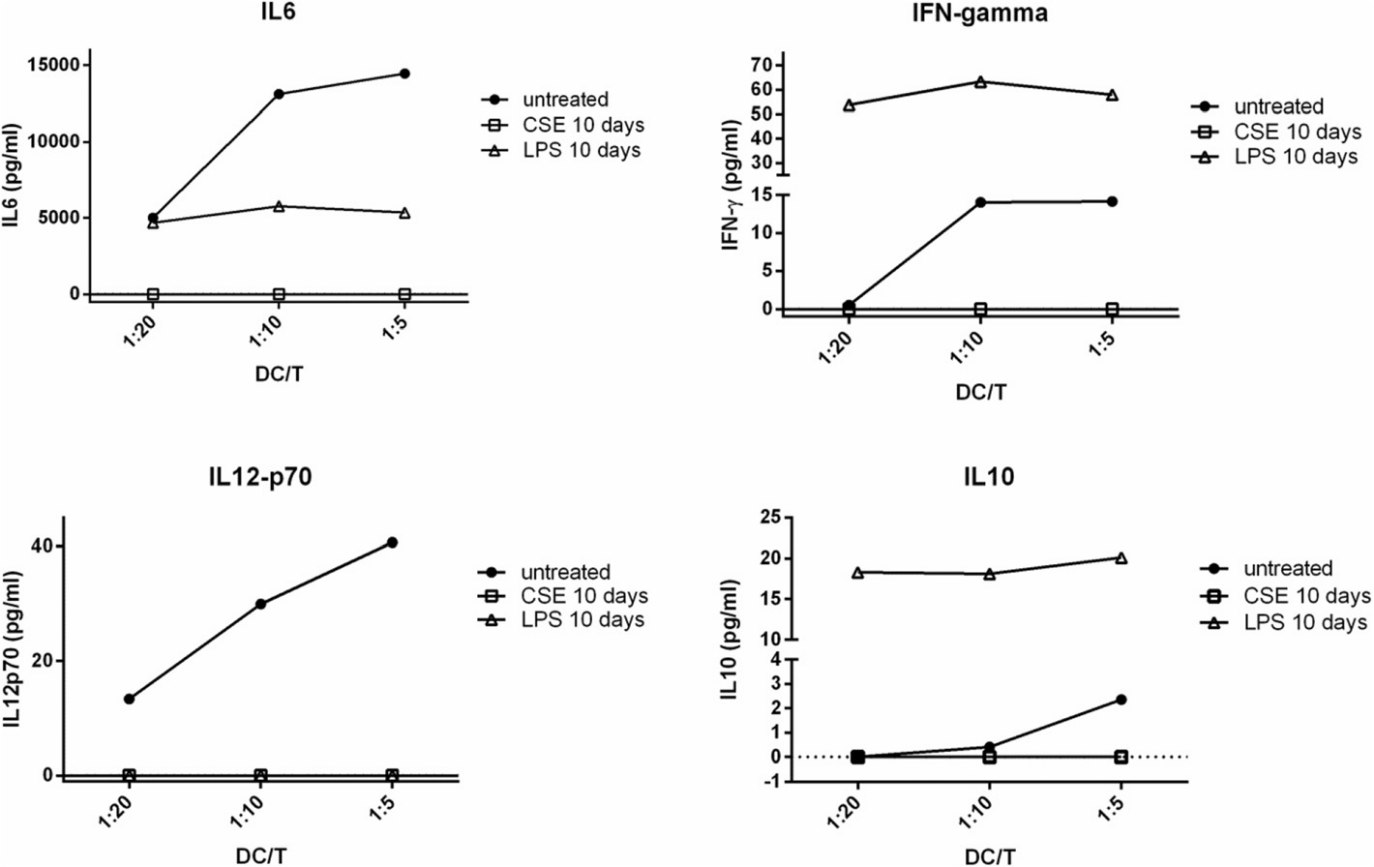
DCs cocultured with CSE for 10d cannot stimulate T cell activation in a mixed lymphocyte reaction. Ovalbumin-specific D011.10 T cells were isolated from spleen and mixed with ovalbumin and bone marrow-cultured DCs (untreated), DCs co-cultured with CSE for 10 days (CSE 10d) or DCs co-cultured for 10 days with LPS (LPS 10 days). Cytokine production was determined in culture supernatant at 72 h

In a mixed lymphocyte reaction with ovalbumin-specific D011.10 T cells, DCs cocultured with CSE for 10 days were not able to stimulate CD4 T cell proliferation (see Additional file 1: Figure S1) and cytokine production. MLR-induced IL-6, IFN-γ, IL12p70 and IL-10 production was virtually absent when CSE-cocultured DCs were mixed with CD4 T cells (Fig. 8). Also proliferation of T cells was greatly reduced with CSE-cocultured DCs (Additional file 1: Figure S1). Notably, LPS co-cultured DCs showed significant IL-6, IL-10 and IFN-γ production. However, this cytokine production was independent of DC:T cell ratio and may be caused by a direct activation of T cells by residual LPS.

### CSE induced suppression of CD54 on human L428 cells in a time-dependent manner

**Fig. 9:**
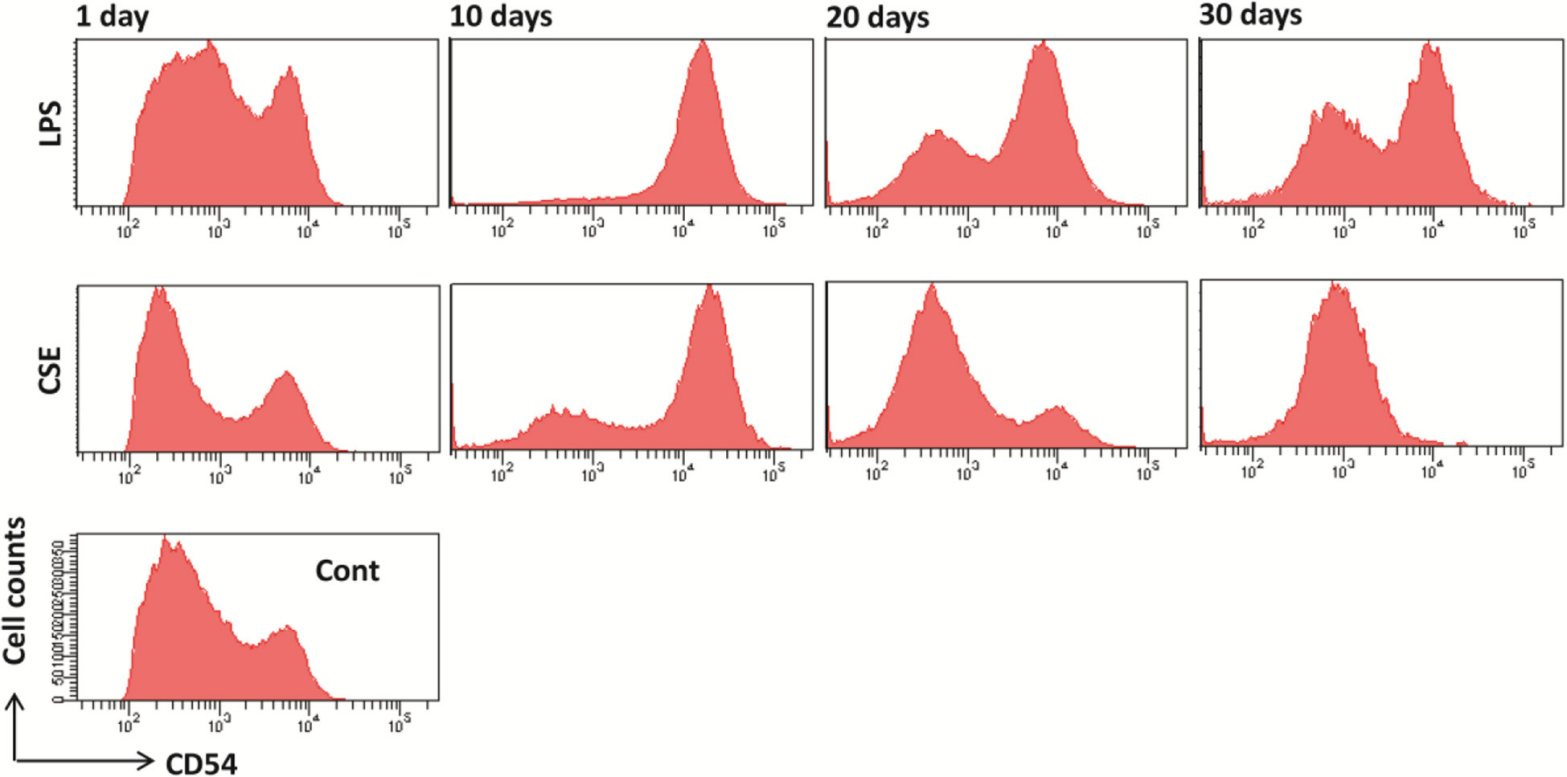
CSE time-dependently suppressed the CD54 expression on human L428 cell line surface. Cells were cultured in presence or absence of CSE (1.5 %) or LPS (100 ng/ml, as positive control) continuously with addition of CSE every day during re-culturing for 10, 20 and 30 days. Representative histograms are showing the cell surface expression of CD54. Data show one representative experiment of three

The human Hodgkin’s disease (HD)-derived cell line L428, closely resembles the human DCs phenotype and function [23, 24]. To determine the effects of CSE on human DCs, L428 cells were incubated with CSE for 10, 20 and 30 days and the expression of CD54 was measured. CD54 is an appropriate marker of APCs as well as an indicator of activation [20]. CSE significantly suppressed the CD54 expression in a time-dependent manner up till day 30 (Fig. 9).

## Discussion

The present study provides evidence that cigarette smoke can directly modulate the DC-mediated immune response by affecting both function and maturation of DCs. We show that similar to LPS acute/short-term coculture with CSE stimulates maturation of newly differentiated and immature DCs, but continuous/long-term exposure to CSE during DC maturation induces “defective and silent” DCs from BM precursors. These DCs are characterized by a down regulation of dendritic cell-specific surface markers, suppressed antigen uptake, and an impaired capacity to stimulate T cells and produce cytokines.

Smoking has been shown to alter a wide range of immunological responses in both man and animal models. The effect of cigarette smoke on DC maturation and function can have important implications in adaptive immune responses in the airways [17, 21, 25]. In the current study, short-term (18–24 h) coculture of DC with CSE stimulated immature DCs towards more mature cells as revealed by upregulation of MHCII, CD83, CD86, CD40, reduction in antigen up-take capacity and enhanced secretion of pro-inflammatory IL-12, IL-6 and TNF-α. These results are in agreement with effects shown after treatment with nicotine [16] and suggest that CSE may drive DCs towards full maturation. Exposure to CSE during late stages of development of DCs (*day* 6) resulted in the full maturation of DC, even to a higher level than could be achieved by LPS. On the other hand, prolonged exposure of BM cells to CSE (for 10 days) causes differentiation of DC precursors into non-functional DCs. Moreover, similar inhibitory effects of long-term co-culture with CSE were found on human L428 cells, which share properties of human DCs resulting in a decreased expression of the activation marker CD54. The phenotypical and functional suppression of DCs induced by CSE was accompanied by reduced expression of maturation markers, impaired capacity to stimulate T cells and produce cytokines. These differential effects to CSE may suggest that timing of exposure during the differentiation of DCs may account for the wide variability observed in studies related to DC maturation and function [14, 16, 26–29].

Our results are in agreement with effects of cigarette smoke on DCs in mouse models and human subjects. For example, Robbins et al that cigarette smoke exposure impairs dendritic cell maturation and T cell proliferation in thoracic lymph nodes of mice. They found that cigarette smoking suppressed DC maturation within the lymph nodes as demonstrated by reduced cell surface expression of MHC class II and the costimulatory molecules CD80 and CD86. DCs from cigarette smoke-exposed animals had a diminished capacity to induce IL-2 production by T cells and was associated with diminished Ag-specific T cell proliferation *in vivo* [30]. Furthermore, our recent *in vivo* experiments showed that modulation of DC subsets in acute and chronic models of cigarette smoke-exposed mice, alters the CS-induce lung inflammation [31]. These findings indicate that cigarette smoke, directly or indirectly, by inducing inflammation and tissue damage can trigger activation and differentiation of DCs. In humans, smoking affects the expression profile of function-associated surface molecules on airway myeloid DCs and induces the recruitment of a large numbers of immature DCs into the small airways of patients with COPD [32–35].

Cigarette smoke contains a complex mixture of chemicals that are capable of exerting immune-modulating effects. *In vitro* studies show that CSE and nicotine have an impact on maturation and function of DCs, which is accompanied with the suppression of chemokine receptor expression and the induction of co-stimulatory receptors. However, reported changes in DC function are not coherent [14, 16, 26–29] and may be related to timing and duration of exposure to cigarette smoke components as evidenced in this study. *In vitro* DC cultures may therefore be useful to gain further insight into the mechanism responsible for the inhibitory effects of cigarette smoke components on DC function and consequently their contribution to the vulnerability of COPD patients to viruses and bacteria.

## Conclusions

Our study shows that cigarette smoke has differential effects on DC’s in vitro. Short term exposure to CSE stimulated maturation of DC generated from mouse bone marrow cells, while long-term co-culture resulted in non-functional DCs with an immature phenotype. Presently, it remains to be investigated if these results can be translated to effects of cigarette smoking in human airways, but it is tempting to speculate that the observed effects may contribute to the vulnerability of COPD patients to viruses and bacteria.

## Additional file

Additional file 1: Figure S1. BMDCs (control, CSE co-cultured, or LPS co-cultured) were mixed with CFSE-labeled DO11.10 T cells (CD4 KJ1-26) in ratio of 1:10 and 1:20 in the presence of OVA peptide for 72 h. After 72 h the CSFE dilution profile were analyzed by flow cytometry. (DOCX 196 kb)

## Abbreviations

APCs: Antigen presenting cells
BM: Bone marrow
COPD: Chronic obstructive pulmonary disease
CS: Cigarette smoke
CSE: Cigarette smoke extract
DC: Dendritic cell
HSC: Hematopoietic stem cell
pre-DCs: Precursors for cDCs
ELISA: Enzyme-linked immunosorbent assay
LPS: Lipopolysaccharide
