# Supplementary material for: Cigarette smoke differentially modulates dendritic cell maturation and function in time
Source: Respir Res. 2015 Oct 24;16:131. doi: 10.1186/s12931-015-0291-6 (PMC4619524; doi:10.1186/s12931-015-0291-6)
Supplement: Additional file 1: Figure S1. — BMDCs (control, CSE co-cultured, or LPS co-cultured) were mixed with CFSE-labeled DO11.10 T cells (CD4 KJ1-26) in ratio of 1:10 and 1:20 in the presence of OVA peptide for 72 h. After 72 h the CSFE dilution profile were analyzed by flow cytometry. (DOCX 196 kb) [file 12931_2015_291_MOESM1_ESM.docx]

Supplemental figure 1


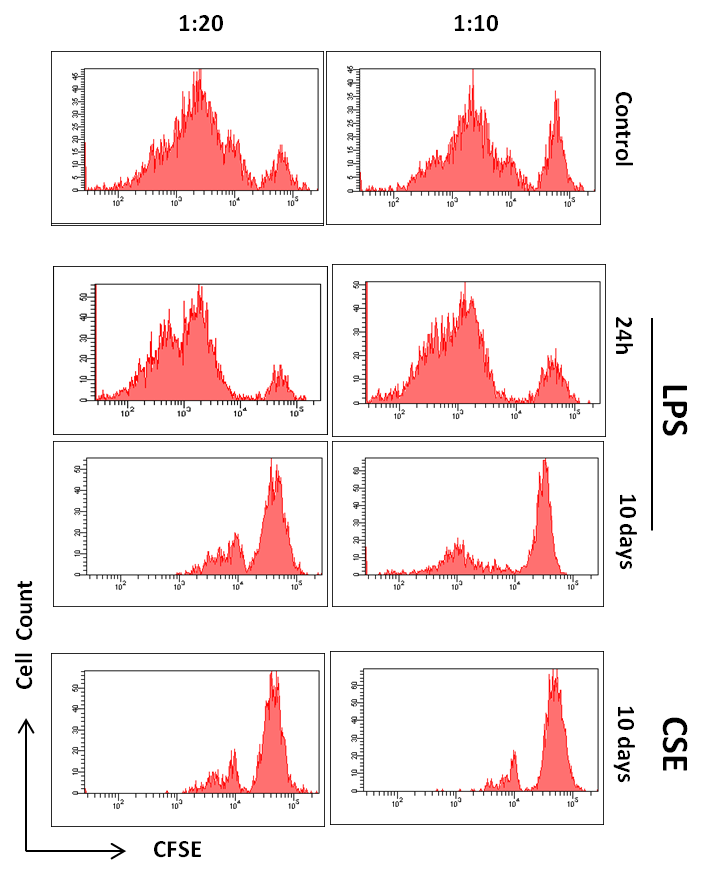


Figure 1. BMDCs (control, CSE co-cultured, or LPS co-cultured) were mixed with CFSE-labeled DO11.10 T cells (CD4 KJ1-26) in ratio of 1:10 and 1:20 in the presence of OVA peptide for 72h . After 72h the CSFE dilution profile were analyzed by flow cytometry.
